# Scattered Light and Other Corrections in Absorption Coefficient Measurements in the Vacuum Ultraviolet: A Systems Approach

**DOI:** 10.6028/jres.095.030

**Published:** 1990

**Authors:** R. Klein, W. Braun, A. Fahr, A. Mele, H. Okabe

**Affiliations:** National Institute of Standards and Technology, Gaithersburg, MD 20899; University of Rome, Pie. A. Moro 5, 00185 Rome, Italy; Howard University, Washington DC 20059

**Keywords:** absorption coefficient, computer automation, error analysis, instrument, vacuum

## Abstract

A systems approach in which computer automation is applied to a vacuum ultraviolet spectrometer and auxiliary components is described. The errors associated with the measurement of gaseous absorption coefficients in the vacuum ultraviolet are considered. The presence of scattered light introduces large errors particularly at those wavelengths where the source used is characterized by low intensity. In the case of a D_2_ light source this occurs in the region 120 to 130 nm. Simple considerations explain the variation of the absorption coefficient determinations in the presence of scattered light and lead to an appropriate treatment of the data to eliminate the error. Experimental results are presented illustrating the efficiency and precision obtainable with the present approach.

## 1. Introduction

Accurate absorption coefficients in the vacuum ultra violet region for a variety of gases are required in many applications such as in the assessment of the photochemistry of man-made halocarbons in the stratosphere [1] and chemical vapor deposition of organometallic compounds [2,3]. The absorption coefficient at a wavelength λ is expressed by the well known relationship

$$\in_{\lambda }=-\left(1/pl\right)\text{ln}\left(I_{\lambda }/I_{0\lambda }\right)$$ (1)

for gases that conform to Beer’s law. It is obvious that the quality of the measurement of the absorption coefficient depends on the precision and accuracy of *p, l, I*, and *I*_0_, the pressure of the gas, length of the absorbing path, and the intensities of the light signals with and without the absorbing gas, respectively. Reference has been made to error analysis in absorption coefficient measurements in the vuv region by Mount et al. [4]. Simon et al. [1] have also considered errors in their investigation of halogenated hydrocarbons in the uv where the values of e are small and difficult to measure. Both Simon et al. [1] and Gillotay et al. [5] presented error budgets based on *p*, *l*, *I*/*I*_0_, *T*, and impurities.

Since *I*_λ_ and *I*_0λ_ in eq (1) are usually determined from separate wavelength scans, the precision in ϵ_λ_ can be adversely affected by lack of wavelength reproducibility, especially in those regions of the spectrum where d*I*_0λ_/dλ is large. Further it is unavoidable that scattered light is included in the measurement of both *I* and *I*_0_, introducing errors in ϵ. The errors can be large depending upon the ratio of the scattered to the transmitted light. Errors in the absorption coefficient measurements must be considered with respect to the specific experimental configuration employed (single beam or double beam, for example). McPherson^1^ provides a dual beam attachment for their monochromators. This device utilizes an oscillating mirror to direct the beam alternately through twin cells, one of which is used for establishing a reference, *I*_r_. The *I* and *I*_0_ signals are obtained using the other cell and individually ratioed with *I*_r_ to remove variations of the resultant ratios (*I*_λ_/*I*_rλ_ and *I*_0λ_*/I*_rλ_) in separate wavelength scans. This technique has been used and discussed by a number of workers [6,7,8]. The measurements described in the present paper are based on a single beam instrument system. High lamp stability and wavelength reproducibility offset the need for a reference beam. We will also show that the presence of a sharp-line spectrum is essential for scattered light diagnosis and correction. In the present work, error analysis for measurements of ϵ in the uv to the vuv will be considered in detail, and a method is developed for obtaining more accurate values of ϵ in regions where scattered light is not neghgible. We have used a systems approach wherein computer control and analysis lead to increased accuracy and efficiency.

## 2. Experimental

**Figure 1:**
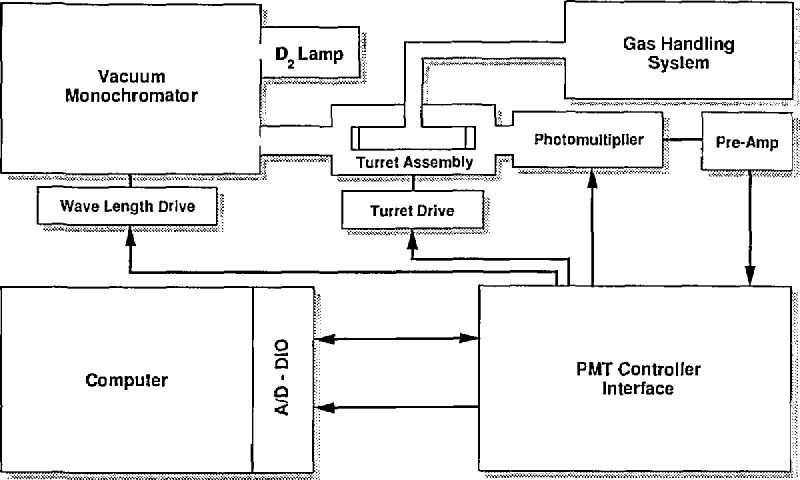
Schematic diagram of the vacuum ultra-violet absorption coefficient acquisition system.

The central part of the system is a 1-m normal incidence McPherson vacuum monochromator employing a 600 lines/mm grating blazed at 150 mn. A Hamamatsu D_2_ lamp with a stabilized power supply is the source of vacuum uv radiation. A MgF_2_ window on the lamp limits the low wavelength cutoff at about 115 nm. The light detector is a solar blind Hamamatsu R1220 side-on photomultiplier tube (PMT) fitted with a MgF_2_ window. The photomultiplier housing [9] permits vacuum operation. Five absorption cells fitted with LiF windows and covering the range from 0.2 to 20 cm in length are arranged in a cylindrical (turret) array so that any one may be interposed in the light path. Two MgF2 windows and two LiF windows establish the lower limit of usable light intensity down to about 119 nm. For the present apparatus the signal to noise ratio of the light signal is about 1*%* (shot noise limited) for integration times of about 1 s [10]. The monochromator wavelength drive is controlled by a stepping motor with 800 steps per revolution. A gear train is employed such that 16 steps are equivalent to 0.1 nm wavelength change. Gas pressure in the cells is monitored with a diaphragm gauge calibrated by an oil manometer. A block diagram of the apparatus is given in figure 1.

The current from the PMT developed by the light signal is processed by a current to voltage pre-amp (with output impedance of 50 Ω) at the PMT. The pre-amp output is connected to a PMT-controller interface module. The PMT-controller interface consists in part of a follower-amp and filter-amp where the signal is amplified by 10 and processed by a programmable voltage amplifier (with binary gain from 2^0^–2^7^. The resulting voltage signal is received by an A/D-DIO (analogue to digital—digital input output) interface board within an IBM compatible PC. The PMT-controller interface contains shaping and latching circuits. These process the digital logic signals (from the A/D-DIO board) used to control the programable amplifier and the stepping motors. The proper functioning of all components can be assessed through a computer test program.

Initially all measurements were made in a single scan, analyzed and displayed in real time. Prior to each run all cells were intercompared (with no sample) to compensate for any imbalance in transmission. At each wavelength an *I*_λ_ and *I*_0λ_ were determined sequentially by rotating the turret from a given filled cell to an empty reference cell. An absorption coefficient was determined to form the basis of a choice of a cell of optimum length (*I*/*I*_0_≈0.5). The wavelength was advanced and the cell of optimum length was then interposed for the measurement. The operation of changing the turret from a given cell to the reference cell and the requirement to measure the cell imbalance at each wavelength added considerably to the effective data acquisition time. The final procedure adopted was simplified as follows. The measurement was performed in two separate scans. The light intensity was monitored with the same cell, first under vacuum and then with the absorbing gas. The data obtained in each scan were stored and later analyzed.

## 3. Analysis of Measurement Errors

From the theory of propagation of errors applied to eq (1), the error in ϵ, is in part

$$\begin{array}{l} d\epsilon_{\lambda }=\left(1/pl\right)[{\left(\text{ln}\left(I_{\lambda }/I_{0\lambda }\right)\right)}^{2}\left\{{\left(dp/p\right)}^{2}+{\left(dl/l\right)}^{2}\right\}\\ {+{\left(dI_{\lambda }/I_{\lambda }\right)}^{2}+{\left(dI_{0\lambda }/I_{0\lambda }\right)}^{2}]}^{1/2}. \end{array}$$ (2)

**Figure 2:**
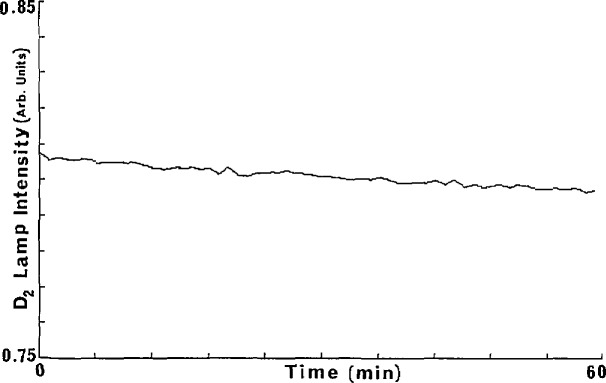
Deuterium lamp stability over a 1-h period.

**Figure 3:**
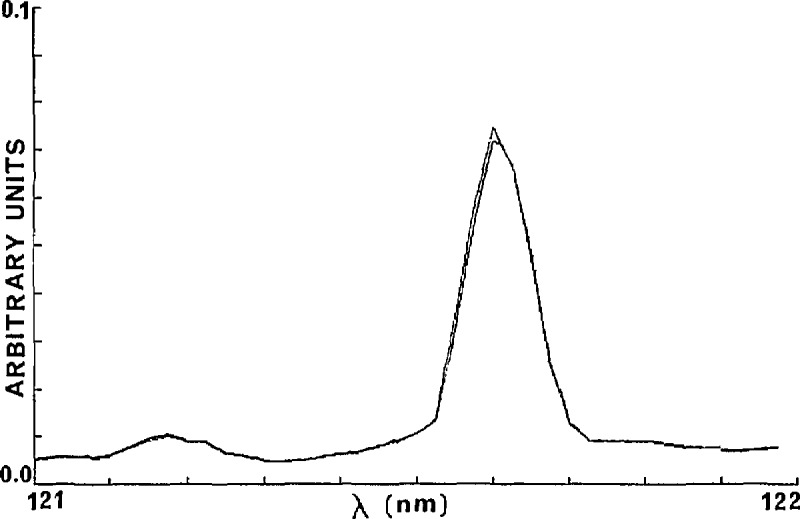
Separate scans of the deuterium lamp demonstrating wavelength reproducibility.

Errors in the pressure and cell length measurements can be held to less than 1 %. In consideration of the measurement system used, the short and long term variations of *I*_0_ need to be examined. A particular *I*_0_ value is obtained from the signal from the PMT. At a given wavelength setting the signal is recorded and stored by way of 1000 A/D conversions. The values, acquired in about 1 s are averaged, and the standard deviation, σ, is calculated. Typical values at 120 nm are *I*_0_=0.2747×lO^−2^±0.2×lO^−3^ and at 161.4 nm *I*_0_=3.3815±0.727×l0^−3^ where the errors represent σ/(l000)^1/2^, which we denote as (σ_av._). This short time fluctuation is small and is entirely associated with shot noise as verified by noting that the ratio σ_av._ (120 nm)/σ_av._ (161.4 nm) is equal to the square root of the ratio of the above two light source signals. The long term stability was determined through measurements over 1-h periods. This is shown in figure 2. Similar drifts in *I* signals are observed. The long term downward drift of about 1% per hour in the light source constitutes a systematic error for which correction can be made. Warm-up periods of at least 20 min are necessary to maintain the level of stability shown in figure 2. As the *I* and *I*_0_ scans are usually taken within minutes the drift shown in figure 2 is usually small enough not to warrant correction. There is an additional error due to scattered light, much larger percentage-wise in *I* than in *I*_0_. This will be discussed in some detail. Another possible source of error is that of the wavelength setting. Even a fraction of an angstrom difference in settings in subsequent determinations involving *I* and *I*_0_ leads to errors, particularly in those regions where d*I*_0_/dλ is large. Because the wavelength drive is controlled by a stepping motor with one pulse equivalent to 0.00625 nm, very precise settings can be achieved, and manual re-settings are eliminated. Since a computer controlled stepping motor is used to advance and rewind the grating drive any irreproducibility in successive wavelength scans is likely to be due to backlash in the connecting gear train. Most of the backlash can be corrected between successive scans by rewinding to below the starting wavelength and then advancing to the starting position. Figure 3 shows two subsequent scans of the lamp spectrum between 121 and 122 nm where the Lyman-α line is the spectral feature. The two curves in this figure are virtually superimposable. It is noted that shifts of even less than one tenth of the spectral line width produces a significant detrimental effect on an absorption coefficient measurement.

Wavelength setting and scattered light errors aside, the percentage error in ϵ is

$$\begin{array}{l} d\epsilon_{\lambda }/\epsilon =[{\left(dp/p\right)}^{2}+{\left(\mathrm{dl}/l\right)}^{2}+{\left(\text{ln}\left(I_{\lambda }/I_{0\lambda }\right)\right)}^{-2}\{{\left(dI_{\lambda }/I_{\lambda }\right)}^{2}\\ {+{\left(dI_{0\lambda }/I_{0\lambda }\right)}^{2}\}]}^{1/2}. \end{array}$$ (3)

**Figure 4:**
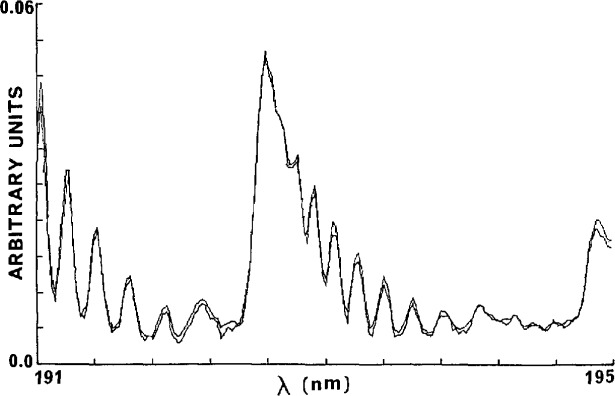
Scans of Schumann-Runge absorption coefficients of oxygen at 79 and 165 Torr using a 20-cm cell demonstrating reproducibility.

As an example, if the percentage errors in *p*, *l*, *I*, and *I*_0_ are taken as 1.0, 0.5, 1.0, and 1.0 with *I*/*I*_0_=0.5, the percentage error in e is about *2*%. This error limit can be achieved routinely, and with extreme care a percentage error of 1% or less can be obtained. Figure 4 shows that in two repeat determinations of the spectrum of oxygen between 190 and 195 nm excellent agreement is observed. This agreement is remarkable in view of the fact that there is only about 1% absorption and hence the stability of the light source must be about 0.1% over the data acquisition time. On the other hand, a comparison of two absorption coefficient determinations using acetone, one with the 0.64-cm cell and the other with a 2.0-cm cell showed a 2% discrepancy. If the 2.0-cm cell were chosen as the standard, the nominal 0.64-cm cell would in fact be 0.653 cm. This is an indication of the sensitivity of the measurements. Finally as far as errors are concerned, it can be inferred from eq (3) that the absorption coefficient measurements are subject to relatively large percentage errors when the absorption, (*I*_0_*—I*)*/I*_0_, is close to zero or one.

## 4. Method of Correction for Scattered Light

**Figure 5:**
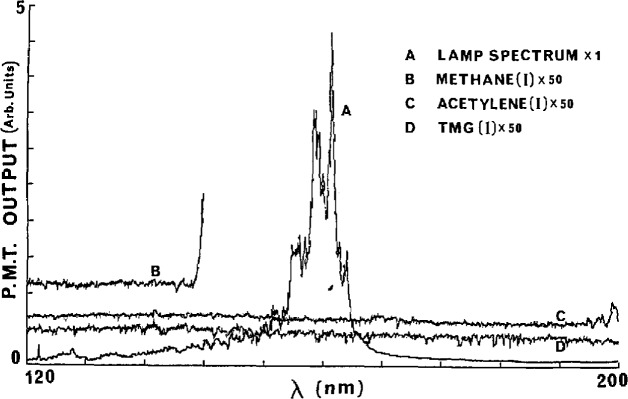
Scattered light from deuterium lamp remaining after absorption by methane, acetylene and trimethyl gallium.

Because of scattered light the efficiency of a grating in isolating a specific wavelength interval is not 100%. For gratings that have deteriorated through long use the efficiency may be considerably less. This presents no difficulty in absorption coefficient measurements where the light intensity is so high that the scattered light is negligible in comparison. Problems are encountered, i.e., obtaining erroneous absorption coefficient values, when the scattered light cannot be ignored. This is the case in the 120–130 nm region of the deuterium lamp spectrum. The presence of scattered light can be convincingly demonstrated through measurements of the transmitted light through a cell filled with an absorbent sufficient to effectively eliminate light at a prescribed wavelength. Scattered light was demonstrated through the use of a 20-cm cell filled with various compounds, acetylene for example, which show high absorption coefficients at wavelengths up to 200 nm. The detector signals are shown in figure 5 for a particular grating. The signals display a non-zero intensity and very little variation over a broad spectral range. Gases absorbing less strongly at longer wavelengths show, as expected, a higher value for the light background.

**Figure 6:**
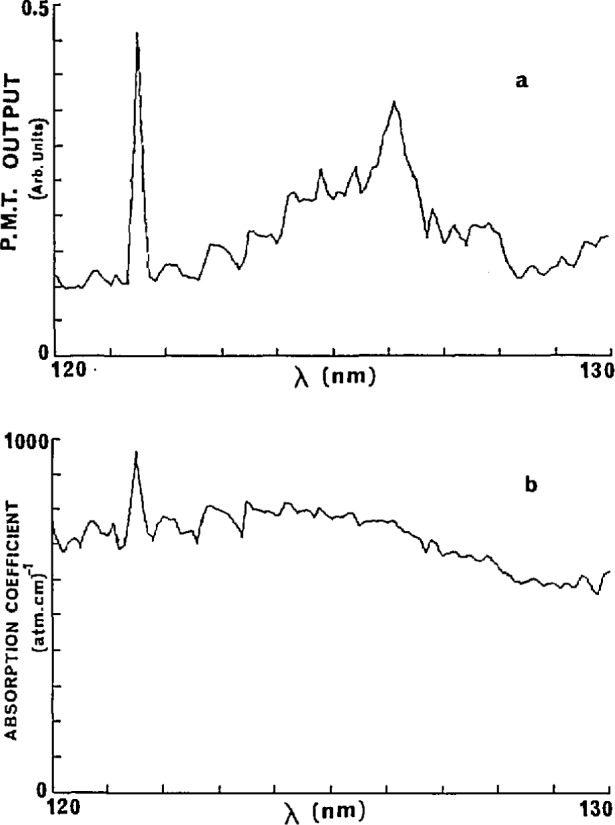
Absorption coefficients of acetone between 120 and 130 nm without scattered light correction showing features of the deuterium lamp spectrum.

The absorption coefficient of acetone between 120 and 130 nm without correction for scattered light is shown in figure 6. It is also noted in figure 6 that the absorption coefficient-wavelength curve has characteristics similar to the lamp spectrum in this wavelength region (but not elsewhere). The basis for this effect, the presence of scattered light which is not an insignificant part of the signal, is examined and an appropriate corrective procedure formulated. That the presence of scattered light can lead to an absorption coefficient spectrum which has superimposed on it certain features of the spectrum of the source lamp is easily shown. If *A* is the scattered light intensity, then the measured absorption coefficient will be of the form

$$\epsilon =-\left(1/pl\right)\text{ln}\left[\left(I+A\right)/\left(I_{0}+A\right)\right]$$ (4)

where ϵ_0_=−(l/*pl*)ln(*I*/*I*_0_). It will be assumed that *A* is negligibly small with respect to *I*_0_. Then −(1/*pl*)ln[(*I*/*I*_0_)(1+*A*/*I*)] can be approximated by

$$\epsilon =\epsilon_{0}-A/\left(plI\right).$$ (5)

*I* is related to the lamp spectrum so that its inverse, subtracted from e_0_, gives an apparent coefficient that has the appearance of an absorption spectrum upon which the lamp spectrum is superimposed. The effect of the scattered light leads to an absorption coefficient that is less than the true value ϵ_0_.

**Figure 7:**
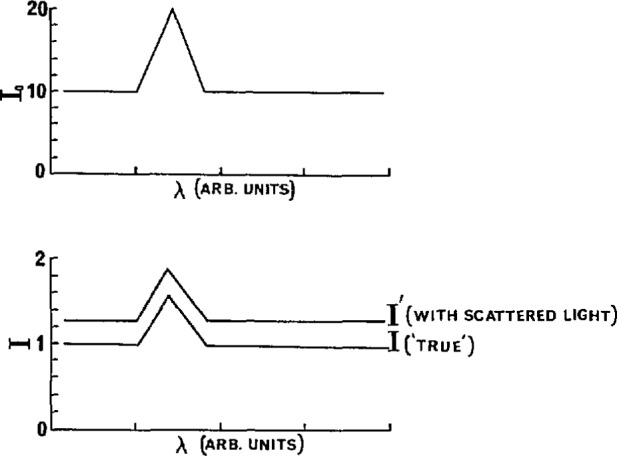
Model for *I* and *I*_0_ with simulated Lyman-α line with and without scattered light.

**Figure 8:**
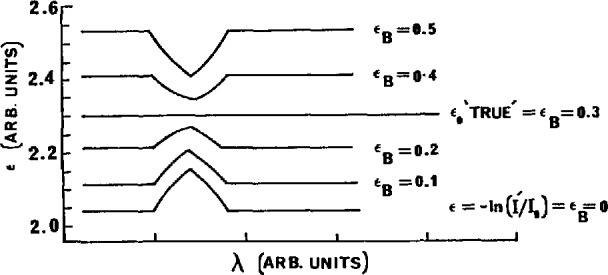
Model, based on figure 7, of the effect of the scattered light correction on the calculated absorption coefficient.

**Figure 9:**
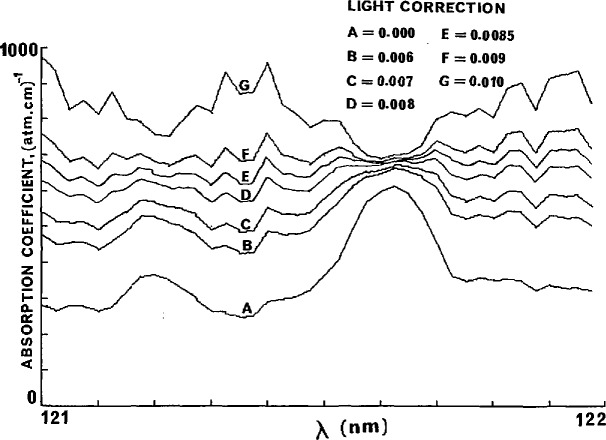
Effect of the scattered light correction on the absorption coefficient of ethane between 121 and 122 nm. Pressure ethane, 2.26 Torr; cell, 0.64 cm.

Obviously the correction for the scattered light requires subtraction from the *measured I* the quantity *A.* Aside from some indication of a minimum of this subtraction, as obtained from figure 5, a criterion must be found to establish the precise value of *A* to be subtracted. The spectral distribution of the scattered light is not known although it is likely to be concentrated in the 160 to 170 nm spectral region, since this is where the D_2_ lamp radiation is most intense. The scattered light is of course attenuated by the presence of the absorbing gas in the cell. The scattered light correction for ethane at 6.59 Torr was found to be 25% lower than that at 0.61 Torr, for example. All the factors for calculating the amount of scattered light ultimately detected by the PMT are not available. Fortunately, the presence of the sharp Lyman-α peak at 121.6 nm in the deuterium lamp spectrum presents an appropriate approach to the solution of the problem. This is illustrated with the following model. It is assumed that over the wavelength region where scattered light may not be neglected, the true absorption coefficient spectrum is taken as constant. The light source spectrum is also assumed constant except for a single triangular spike (approximating the Lyman-α line). A value of 10 is assigned to *I*_0_, 1.0 to *I*, and 0.3 to *A*, the scattered light, and 1.0 to the product *pl* (fig. 7). If *I*′ refers to the directly observed value of the intensity after absorption then *I* is *I′—A.* A corrective term *B* applied to *I′* will give ϵ=ϵ_0_*—C/*(*plI*) where *C=A—B* will be positive, zero, or negative depending on the choice of *B.* Figure 8 illustrates each of these cases. The criterion for the correct value of *B* is the complete elimination of the spike. The validity of this model is shown in figure 9 where the absorption spectrum of ethane was established over the 121–122 mn region. An appropriate value of 5 was found, and hence an accurate value of ϵ_0_ could be established.

It can be seen from figure 9 that if no scattered light correction is made there is about 15% error at the Lyman-α peak and more than 100% elsewhere in the 121–120 range. This is in accord with the expression derived from eq (3), namely dϵ/ϵ=(*A*/*I*)/ln(*I*/*I*_0_), and with about 5% and 50% scattered light on and off the Lyman-α peak respectively [6]. We have also found that an estimate for the correction obtained from the residual light through a high pressure absorbing gas is not appropriate and is generally about half that derived by the procedure shown in figure 9.

## 5. Summary

An automated control and data acquisition system for the measurement of absorption coefficients in the vuv region of the spectrum is described. Further, an error analysis for these measurements has been made. It shows that besides the obvious contributions of errors in the pressure and cell length determinations, scattered light contributes to the error budget especially in regions of low light source intensities. Corrections for the latter can be made if a sharp line in the source, such as Lyman-α in the D_2_ lamp output, is present. System automation permits the achievement of precise wavelength reproducibility and fast data acquisition, processing and recording. The system approach described here for obtaining absorption coefficients in the vuv is shown to provide an improvement not only in the accuracy and precision but also in the efficiency with which these measurements can be made.

## Footnotes

1 Certain commercial equipment, instruments, or materials are identified in this paper to specify adequately the experimental procedure. Such identification does not imply recommendation or endorsement by the National Institute of Standards and Technology, nor does it imply that the materials or equipment identified are necessarily the best available for the purpose.
